# Regulation of Coronary Blood Flow by the Carotid Body Chemoreceptors in Ovine Heart Failure

**DOI:** 10.3389/fphys.2021.681135

**Published:** 2021-05-28

**Authors:** Mridula Pachen, Yonis Abukar, Julia Shanks, Nigel Lever, Rohit Ramchandra

**Affiliations:** ^1^Department of Physiology, University of Auckland, Auckland, New Zealand; ^2^Department of Medicine, University of Auckland and Green Lane Cardiovascular Service, Auckland City Hospital, Auckland, New Zealand

**Keywords:** heart failure, coronary blood flow, carotid body, chemoreflex activation, cardiorespiratory, sympathetic nerve activity

## Abstract

Carotid bodies (CBs) are peripheral chemoreceptors, which are primary sensors of systemic hypoxia and their activation produces respiratory, autonomic, and cardiovascular adjustments critical for body homeostasis. We have previously shown that carotid chemoreceptor stimulation increases directly recorded cardiac sympathetic nerve activity (cardiac SNA) which increases coronary blood flow (CoBF) in conscious normal sheep. Previous studies have shown that chemoreflex sensitivity is augmented in heart failure (HF). We hypothesized that carotid chemoreceptor stimulation would increase CoBF to a greater extent in HF than control sheep. Experiments were conducted in conscious HF sheep and control sheep (*n* = 6/group) implanted with electrodes to record diaphragmatic electromyography (dEMG), flow probes to record CoBF as well as arterial pressure. There was a significant increase in mean arterial pressure (MAP), CoBF and coronary vascular conductance (CVC) in response to potassium cyanide (KCN) in both groups of sheep. To eliminate the effects of metabolic vasodilation, the KCN was repeated while the heart was paced at a constant level. In this paradigm, the increase in CoBF and CVC was augmented in the HF group compared to the control group. Pre-treatment with propranolol did not alter the CoBF or the CVC increase in the HF group indicating this was not mediated by an increase in cardiac sympathetic drive. The pressor response to CB activation was abolished by pre-treatment with intravenous atropine in both groups, but there was no change in the CoBF and vascular conductance responses. Our data suggest that in an ovine model of HF, carotid body (CB) mediated increases in CoBF and CVC are augmented compared to control animals. This increase in CoBF is mediated by an increase in cardiac SNA in the control group but not the HF group.

## Introduction

Heart failure (HF) is a severe, debilitating condition with poor survival rates and increasing prevalence (Swedberg et al., 2005). Amongst other features, patients with HF commonly exhibit disordered breathing patterns and autonomic dysfunction (Chua et al., 1996, 1997). A characteristic of the autonomic dysfunction is an increase in sympathetic nerve activity to the heart which acutely provides inotropic support and maintains cardiac output (Watson et al., 2006) but over the long-term also promotes disease progression and shortens life expectancy (Kaye et al., 1995). Both the disordered breathing and the autonomic dysfunction are strongly related to a higher mortality risk and poorer prognosis in patients (Cohn et al., 1984; Floras, 1993; Naughton et al., 1993; Leung et al., 2004). Previous studies have suggested that altered neural reflexes, such as the chemoreflex and baroreflex contribute to the elevated levels of sympathetic nerve activity in HF (Ferguson et al., 1992; Ponikowski et al., 1997; Narkiewicz et al., 1998, 1999).

Recently, the carotid body (CB) has been implicated as playing a major role in mediating the increase in sympathetic nerve activity during cardiovascular disease. The CB is a polymodal chemoreceptor strategically located at the bifurcation of the common carotid artery. Amongst other stimuli, the CB is activated by hypoxia, hypercapnia, and acidosis (Iturriaga et al., 2016). Studies have shown that the sensitivity of the chemoreflex is elevated in patients with HF (Chua et al., 1997; Schultz and Li, 2007) and animal models of HF (Sun et al., 1999; Del Rio et al., 2013). Interrupting the chemoreflex both acutely using intranasal oxygen (Xing et al., 2014) and chronically using denervation of the CB (Marcus et al., 2014) has been shown to reduce sympathetic nerve activity to the heart and the kidney.

The primary blood supply to the heart has been less studied in both control and disease conditions. Oxygen extraction is already high in the heart (Binak et al., 1967) which means that energy demand is the primary regulator of coronary blood flow (CoBF). However, neural control of the coronary artery resistance has also been shown to play an important role. In this context, our previous study in conscious sheep showed that activation of the CB increased directly recorded cardiac SNA and CoBF (Pachen et al., 2020). Importantly when the effects of sympathetic activation was blocked, the increase in CoBF was abolished indicating activation of the chemoreflex increases cardiac SNA which augments CoBF (Pachen et al., 2020).

There is profound activation of cardiac SNA in animal models of heart failure (Ramchandra et al., 2008, 2009) and this increase is at least partly mediated by the CB chemoreflex (Xing et al., 2014). However, the role of the CB chemoreceptors in regulating CoBF in HF remains unclear. Therefore, the first aim of our study was to examine if the CoBF response to CB activation is altered in HF. We hypothesized that CB activation would result in an attenuated increase in CoBF in conscious animals with HF. We also hypothesized that inhibition of the sympathetic activation to the heart using a beta-blocker would attenuate the increase in CoBF.

## Methods

Experiments were conducted on conscious, adult female Romney sheep weighing 50–80 kg, housed in individual crates and acclimatized to laboratory conditions (18°C, 50% relative humidity, and 12 h light-dark cycle) and human contact before experimentation. The sheep were fed 2 kg/day (Country harvest pellets), water *ad libitum* and supplemental hay or chaff as needed. All experiments and surgical procedures were approved by the Animal Ethics Committee of the University of Auckland.

### Heart Failure Induction

To induce HF, animals had microspheres infused into their coronary arteries as described previously (Abukar et al., 2019). Briefly, sheep were fasted for 24 h prior to surgery and anesthesia was induced with intravenous propofol (5 mg/kg, i.v.; AstraZeneca, United Kingdom), and following intubation maintained with 1.5–2.0% isoflurane/oxygen (O2; Lunan Better Pharmaceutical, China). Once anesthetized and intubated, the sheep were placed in a supine cradled position and four limb electrodes were inserted to record ECG. The left or right femoral artery was accessed percutaneously with an 8F (CORDIS^®^, United States) sheath. The left main coronary artery was then cannulated using an 8F AL2 (CORDIS^®^, United States) guide catheter under fluoroscopic guidance. HF was induced by infusion of polystyrene latex microspheres (45 microns; 1.3 ml, Polysciences, Warrington, PA, United States) into the left coronary artery, with three sequential embolizations over 3 weeks, to ensure maximum left ventricle coverage. Prior to each embolization procedure, β-blocker (metoprolol up to 20 mg/kg, IV) and Xylocaine (2 mg/kg, IV) were injected intravenously in order to reduce acute inflammation and prevent ventricular arrhythmias.

Microembolized sheep underwent transthoracic left ventricular echocardiography when the sheep were back in the lab after the holding time (8–10 weeks after the last embolization). The development of HF was assessed by measurement of ejection fraction and fractional shortening by using short axis M-wave echocardiography on conscious sheep standing on their crate. Once sheep were deemed to have sufficient left ventricular dysfunction (ejection fraction < 45%), instrumentation surgery (thoracotomy) was performed.

### Surgery and Anesthesia Protocol

For these studies, a group of HF (*n* = 6) and control (*n* = 6) adult female sheep (weight: 65 ± 10 kg) were used. There was no difference in age between the two groups of animals. Both groups of animals underwent the same instrumentation surgeries. General anesthesia administered by an experienced animal technician. Sheep were fasted for 24 h prior to surgery and anesthesia was induced with intravenous propofol (5 mg/kg, i.v.; AstraZeneca, United Kingdom), and following intubation maintained with 1.5–2.0% isoflurane/oxygen (O2; Lunan Better Pharmaceutical, China). Bupivacaine-Claris solution (2.5 mg/Ml, i.m.; Multichem NZ Ltd., New Zealand) injection was given into the second to sixth intercostal spaces as a nerve block to prevent surgical pain. Ketoprofen (2 mg/kg, i.m.; Merial, Boehringer Ingelheim, NZ) and long-acting Oxytetracycline (20 mg/kg i.m.; Oxytetra, Phenix, NZ) were used for premedication for all sheep.

### Surgical Instrumentation

After thoracotomy, a small area of the proximal left circumflex artery was dissected free and a size 6 transonic flow probe (6PS, Transonic Systems, United States) was positioned around the vessel to measure coronary flow. A size 28 transonic flow probe (28PS, Transonic Systems, United States) was also placed around the ascending aorta to measure cardiac output (CO). Two strips of seven-stranded Cooner Wires (AS 633-7SSF, Cooner Wire, CA, United States) were implanted into the diaphragm and secured with silicone gel to measure dEMG. A pacing lead was also attached to the left atrium to enable pacing of the heart. Following this, the incision site was closed and negative pressure restored to the thoracic cavity.

During the same surgery, an incision was made in the neck to expose the common carotid artery and the jugular vein. A cannula was inserted into the common carotid artery (with the tip of the cannula lying 1 cm proximal to the carotid sinus region) and jugular vein for the drug and saline infusion. In addition, a sterile solid-state pressure catheter (Mikro-Tip, Millar, United States) was inserted in the same carotid artery but toward the heart for the measurement of arterial blood pressure.

### Experimental Protocols

The experiments were performed on conscious sheep at least 48 h after the instrumentation surgery. After recording 30 min of baseline cardiovascular variables, the chemoreceptor reflex response was assessed using KCN (Sigma-Aldrich, Germany) dissolved in 0.9% sodium chloride solution (Baxter, United States) injected as an intra-carotid bolus dose (via the common carotid cannula) at 10, 20, and 30 μg/kg followed by 5 ml heparinized saline (25 IU heparin in 1L saline). Before each set of KCN injection was carried out, 5 ml of saline at room temperature was injected into the carotid artery as a control and the effects on hemodynamic parameters were examined. To eliminate the contribution of altered metabolic demand on CoBF, the heart was paced at a constant rate. The left atrium was paced at a constant rate 10–15 bpm higher than the spontaneous sinus rate using a Grass stimulator (Grass SD9 stimulator; Grass Instruments, United States).

The hemodynamic response to CB activation were determined following cholinergic blockade with intravenous atropine (8 mg bolus followed by 24 mg/h infusion for 30 min; LKT Labs, United States) and β-adrenergic receptor blockade with propranolol (30 mg bolus followed by 0.5 mg/kg/h infusion for 90 min; Merck, Germany). Following drug infusion, the effect of CB activation was repeated with the three KCN doses in all studies. For the study of propranolol and atropine effects, comparisons were made with pacing done at the same level of HR in both control and drug conditions.

The response of cardiovascular variables to CB activation was determined as absolute changes of MAP and breathing response in response to intra-carotid KCN (10, 20, and 30 μg/kg) injection. Each KCN injection was given at least 2 min apart and when HR and BP were stable. Care was taken to avoid any distraction which may have caused changes in baseline recording. To determine the effect of CB activation, the absolute change of each cardiovascular variable in response to intra-carotid KCN over a 60 s period was compared to its respective 15-s average immediately before the injection of KCN bolus. The KCN effect was short-lasting (<60 s), and repeatable. At the end of these experiments, sheep were euthanized with an overdose of intravenous sodium pentobarbitone (7.5 g; Provet NZ Pty Ltd., New Zealand).

### Hemodynamic Recordings

MAP was obtained by connecting the carotid artery pressure catheter to a pressure control unit (Millar, pressure control unit, United States); the pressure catheter was connected to the transducer to check the zero point and the value was noted before the surgery. CoBF (1,000 Hz), MAP (100 Hz), and CO (1,000 Hz) were recorded on a computer using a CED micro 1,401 interface and Spike 2 software (Cambridge Electronic Design, Cambridge, United Kingdom). Heart rate (HR) was measured from the AP signal. The dEMG electrodes were amplified and band-pass filtered (0.3–3.0 kHz). The analog output was fed into a computer data sampling system (CED Model 1401, Cambridge Electronics Design) and processed by a signal analysis program (Spike 2, Cambridge Electronics Design).

### Hemodynamic Measurements and Analysis

Data were analyzed on a beat to beat basis using custom-written routines in the Spike 2 program. For each heartbeat, the program determined diastolic, systolic, MAP, HR, CoBF, CO and the area of the rectified and integrated (time constant = 20 ms) dEMG signals between diastolic pressures. MAP, CoBF, CO, and HR were obtained from the recording and 5 s averages were obtained at 5 s intervals and were presented as a time series graph over 75 s for each animal. The respiratory rate after KCN infusion was calculated manually by visual inspection of the raw dEMG signal. The rectified and integrated dEMG signal with a low-pass time constant of 20 ms was computed using a script on Spike2. Data were expressed as change in absolute values with the resting value being that in the 15 s prior to KCN infusion. Furthermore, CVC (mL/min/mmHg) was calculated by dividing CoBF by the MAP for each heartbeat.

### Statistical Analysis

Results are expressed as mean ± SD. All statistical analysis was done using SPSS software (SPSS, IBM, New York, NY). For baseline KCN data, an unpaired *t*-test was used to compare normal vs. HF animals. Unpaired *t*-tests were used to compare differences in resting hemodynamic variables in response to intravenous drug infusion. The KCN data were assessed using a two-way ANOVA. For time-series data, the dependent variables were MAP, CoBF, CO, CVC, dEMG, and HR. Simultaneously, the independent fixed factors are time, animal and drug treatment (KCN dose, receptor blockade as relevant). The effects of time, drug, and the interaction effect was examined. A significant result was considered if *P* < 0.05.

## Results

### Resting Hemodynamic and Cardiovascular Variables

The resting levels of hemodynamic and cardiovascular variables in the control and HF sheep are shown in Table 1. The microembolization procedure resulted in significant decrease in left ventricular ejection fraction (*p* < 0.001) and fractional shortening (*p* < 0.0001) in HF sheep, after 12–14 weeks. This was associated with a significant decrease in baseline CO compared to control animals. There were no differences in MAP, CoBF, and CVC between the groups (Table 1).

**TABLE 1:** Resting values for hemodynamic parameters between conscious normal and heart failure sheep.

	Basal	
	
	Control	Heart failure
Ejection fraction (%)	793	395*
Fractional shortening (%)	432	202*
Mean arterial pressure (mmHg)	7915	789
Coronary blood flow (mL/min)	7637	7243
Heart Rate (bpm)	1018	9510
Coronary vascular conductance (mL/min/mmHg)	10.7	0.80.5
Cardiac output (L/min)	9.61.4	7.61.3*
Heart mass (g)	36420	43028

Data are represented as mean ± SD. P ≤ 0.05 (unpaired t-test). *Denotes significantly different from basal values. n = 6/group.

### Hemodynamic and Breathing Responses to CB Activation

**FIGURE 1:**
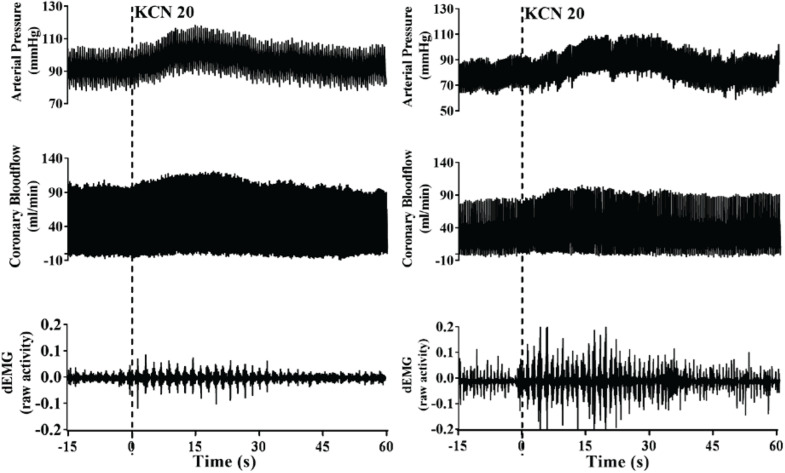
Representative raw traces from one control animal (left) and one heart failure animal (right) demonstrating changes in arterial blood pressure, coronary blood flow, and dEMG in response to intra-carotid potassium cyanide at a dose of 20 μg/kg.

**FIGURE 2:**
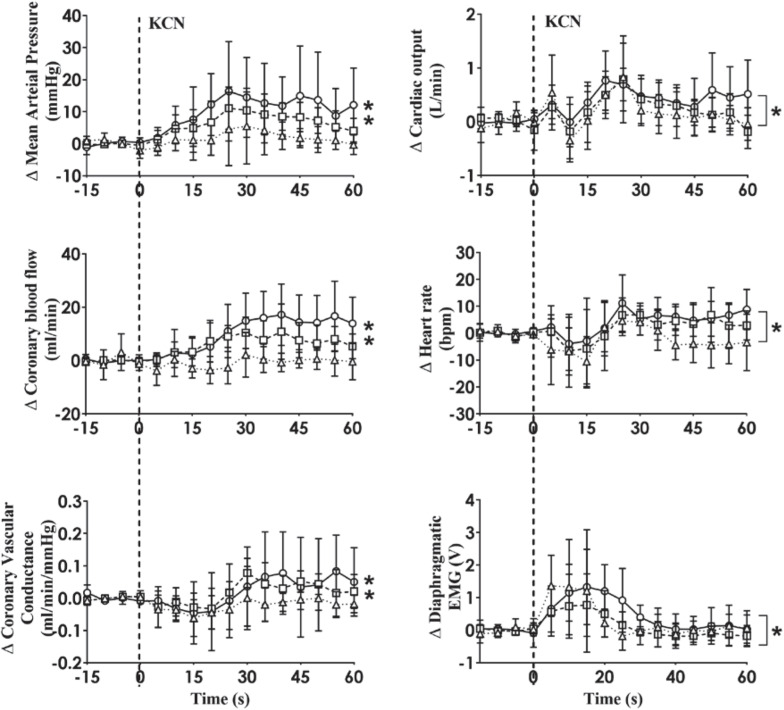
Absolute change in mean arterial pressure, coronary blood flow, coronary vascular conductance, cardiac output, heart rate and dEMG responses (*n* = 6) to intra-carotid potassium cyanide (KCN; 10 μg/kg (triangles and dotted line), 20 μg/kg (squares and interrupted line), and 30 μg/kg (circles and solid line) injection in sheep with HF. Data are mean ± SD. *p < 0.05.

A representative raw data trace from a single control and HF animal demonstrating changes in arterial pressure, CoBF, and dEMG in response to intra-carotid KCN (20 μg/kg) are shown in Figure 1. KCN injection resulted in a significant increase in MAP, CoBF, and dEMG in both groups. KCN at 10, 20, and 30 μg/kg caused a dose-dependent increase in MAP, CoBF, CVC, CO, HR, and dEMG (all *p* < 0.05) when the heart was not paced (Figure 2). There was no significant change in MAP, CoBF, and CVC in response to the lowest dose of KCN (10 μg/kg).

**FIGURE 3:**
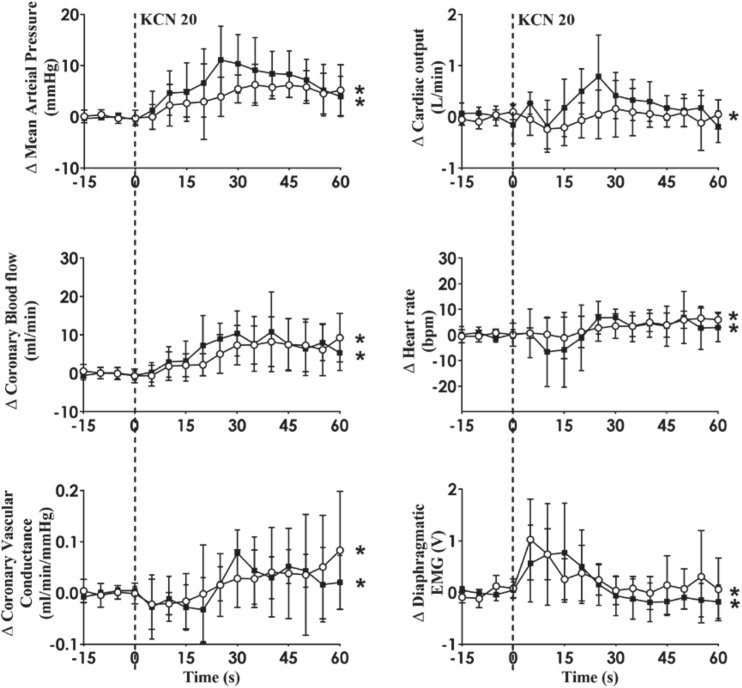
Effect of carotid body activation on breathing and hemodynamic responses in control (*n* = 6) and heart failure (HF; *n* = 6) sheep. Absolute change in mean arterial pressure, coronary blood flow, coronary vascular conductance, cardiac output, heart rate, and dEMG in response to intra-carotid potassium cyanide (KCN; 20 mg/kg) injection in control (open circles and solid line) and heart failure (filled squares and solid line) sheep. Results are mean SD. *p < 0.05 denotes a significant effect of time (ANOVA, *effect within 1 min time after injections).

**FIGURE 4:**
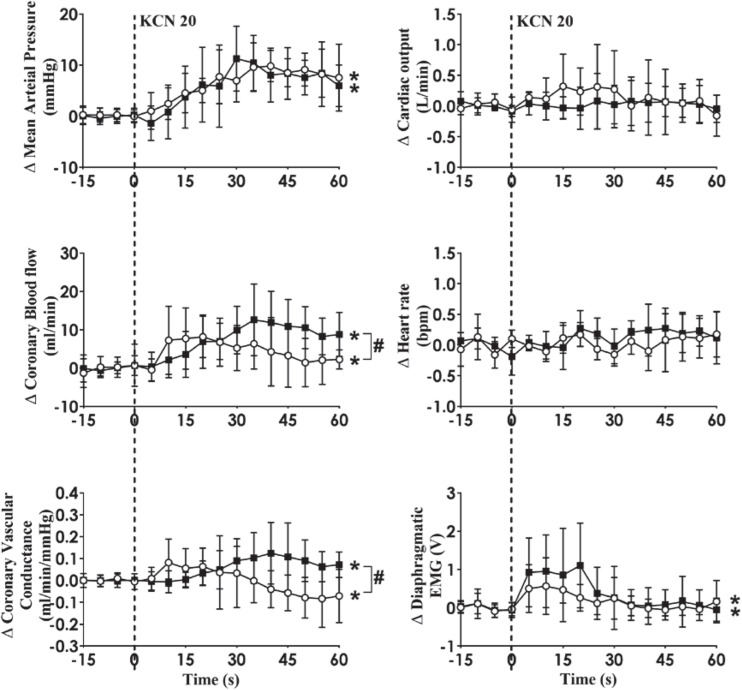
Absolute change in mean arterial pressure, coronary blood flow, coronary vascular conductance, cardiac output, heart rate, and dEMG in response to intra-carotid potassium cyanide (KCN; 20 mg/kg) injection in control (*n* = 6) and heart failure (*n* = 6) sheep. The heart was paced at a constant rate such that the heart rate was unchanged. The open circles and solid line represent control, and filled squares and solid line represents HF animals. Results are mean ± SD. *p < 0.05 denotes a significant effect of time (ANOVA, *effect within 1 min time after injections; ^#^significant interaction effect of time × group).

Intra-carotid KCN infusion at 20 μg/kg led to similar increases in MAP, CoBF, and CVC. Importantly there was a significant increase in heart rate in both groups and a significant increase in CO in the HF group (Figure 3). To eliminate the effects of the increase in heart rate and CO which would have activated metabolic vasodilation, the heart was then paced and the effects compared between both groups. Activation of the CB led to an increase in MAP, CoBF, CVC, and dEMG in both groups (*p* < 0.05; Figure 4). Interestingly, the increase in CoBF and CVC was significantly greater in the HF group than the control group (*p* < 0.05, interaction term). The increase in CoBF in the control animals occurred within the first 15 s of the KCN bolus injection. In contrast, in HF, the CoBF gradually increased after the bolus KCN injection and persisted for a minute. There was no significant difference in the ventilatory response to intra-carotid KCN between the control and HF sheep. Furthermore, there was no change in either HR or CO in either group when the heart was paced (Figure 4).

### Effect of Atropine on the Cardiovascular Variables to CB Stimulation

**FIGURE 5:**
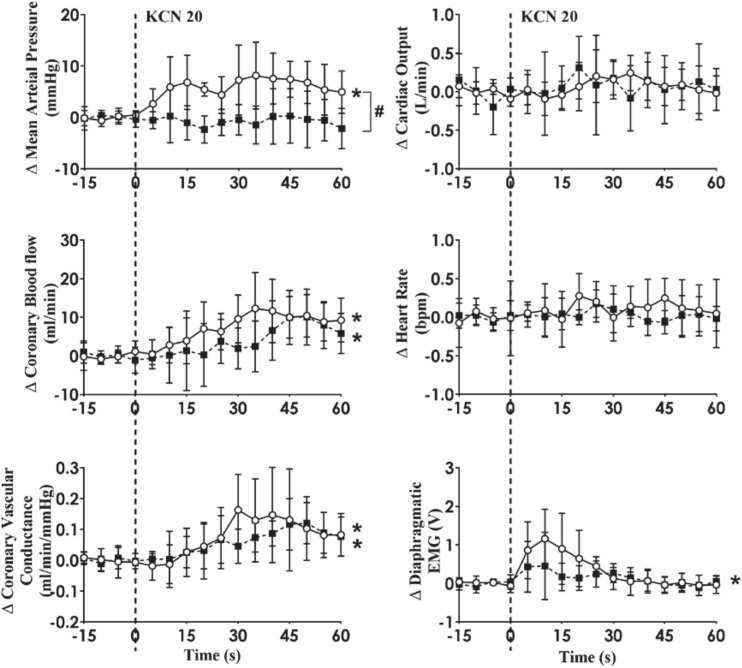
Absolute change in cardiovascular variables to carotid body activation before and after muscarinic receptor blockade (Atropine sulfate; 8 mg bolus followed by 24 mg/h infusion for 30 min) in conscious sheep with heart failure. The heart was paced at a constant rate such that the heart rate was unchanged. The open circles and solid line represent before atropine, and filled squares and interrupted line is after atropine responses. Results are mean ± SD. *p < 0.05 denotes a significant effect of time (ANOVA, *effect within 1 min time after injections; ^#^significant interaction effect of time × group).

As shown in Table 2, atropine infusion resulted in significant hemodynamic changes in control and HF sheep (Table 2) such as increased basal MAP (control: from 76 ± 15 to 86 ± 14 mmHg; *p* < 0.05, HF: from 76 ± 8 to 84 ± 9 mmHg; *p* < 0.05) and HR (control: from 107 ± 4 to 124 ± 16 bpm; *p* < 0.05, HF: from 84 ± 9 to 111 ± 15 bpm; *p* < 0.05). All the other variables were unchanged after atropine infusion. Following intravenous atropine infusion, the MAP response to intra-carotid KCN injection was significantly attenuated (*p* < 0.05), but no other variables were altered (Figure 5).

**TABLE 2:** The effect of intravenous atropine infusion on resting hemodynamic variables.

	Control		Heart failure	
		
	Basal	After atropine	Basal	After atropine
Mean arterial pressure (mmHg)	7615	8614*	768	849*
Coronary blood flow (mL/min)	7937	8841*	8240	9568
Heart rate (bpm)	1075	12416*	849	11115*
Coronary vascular conductance (mL/min/mmHg)	10.7	10.6	0.80.5	0.90.7
Cardiac output (L/min)	9.61.4	8.62.3	7.61.3	7.52.5

The left and right panels panel indicate the effect of intravenous atropine infusion on resting haemodynamic variables in control and heart failure sheep respectively. Data are represented as mean ± SD. P ≤ 0.05 (paired t-test). *Denotes significantly different from basal values.

### Effect of Propranolol on the Cardiovascular Variables to CB Stimulation

Infusion of propranolol significantly decreased basal MAP in both groups (control: from 77 ± 15 to 70 ± 14 mmHg; *p* < 0.05, HF: from 86 ± 14 to 79 ± 17 mmHg; *p* < 0.05) as shown in the first (control) and second (HF) column of Table 3. There was a significant decrease in HR in the HF group while there was no significant change in HR in the control group (interaction effect, *p* < 0.05).

**TABLE 3:** The effect of intravenous propranolol infusion on resting hemodynamic variables.

	Control		Heart failure	
		
	Basal	After propranolol	Basal	After propranolol
Mean arterial pressure (mmHg)	7715	7014*	8614	7917*
Coronary blood flow (mL/min)	8232	8129	7532	7127
Heart rate (bpm)	1018	9610	978	884*
Coronary vascular conductance (mL/min/mmHg)	10.7	10.6	0.80.6	0.70.6
Cardiac output (L/min)	9.41.5	9.41.5	7.21.2	8.12.0

The left and right panels indicate the effect of intravenous propranolol infusion in control and heart failure sheep respectively. Data are represented as mean ± SD. P ≤ 0.05 (paired t-test). *Denotes significantly different from basal values.

**FIGURE 6:**
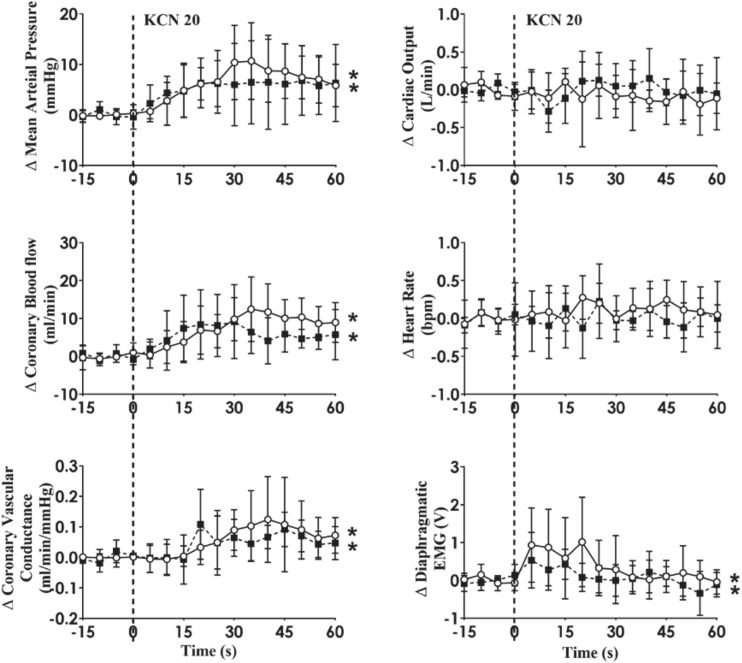
Absolute change in hemodynamic variables to CB activation before and after b-adrenergic receptor blockade (Propranolol; 30 mg bolus followed by 0.5 mg/kg/h infusion for 90 min) in conscious sheep with heart failure. The heart was paced at a constant rate such that the heart rate was unchanged. The open circles and solid line represent the KCN response before propranolol infusion, and filled squares and interrupted line is after propranolol infusion. Results are mean ± SD. *p < 0.05 denotes a significant effect of time (ANOVA, *effect within 1 min time after injections).

In HF, KCN injection resulted in significant increases in MAP, CoBF, CVC, and dEMG (open circles and solid line; all *p* < 0.05). Following intravenous propranolol infusion, there were no significant differences in any of the measured variables in response to KCN injection during pacing condition (Figure 6).

## Discussion

Our study is one of the first to investigate the regulation of CoBF by the carotid bodies in an animal model of HF. The main findings from this study are: (1) Activation of the carotid bodies increases CoBF and CVC in both normal and HF sheep. The increase in both CoBF and CVC is significantly enhanced in the HF group compared to the control group. (2) Inhibition of muscarinic mechanisms using atropine abolished the MAP response to CB activation in heart failure similar to that reported in control animals previously. (3) The increase in CoBF and CVC during activation of the CB is not mediated by elevated sympathetic activity in the HF group, in contrast to control animals.

### The Arterial Pressure Responses to CB Activation in Control and HF

The mammalian CB consists of clusters of parenchymal chemoreceptor glomus (type I) cells which are innervated through the carotid sinus nerve by sensory afferent nerve terminals whose chemo-afferent cell bodies lie in the petrosal ganglion (Gonzalez et al., 1994). The sensitivity of the chemoreflex has been shown to be elevated in patients with HF (Schultz and Li, 2007) and animal models of HF (Sun et al., 1999; Del Rio et al., 2013) and interruption of the chemoreflex (Marcus et al., 2014; Xing et al., 2014) reduces sympathetic nerve activity to the heart and the kidney and also improves autonomic dysfunction and ventilatory instability (Marcus et al., 2014; Xing et al., 2014). We have previously shown that activation of the CB in the conscious state leads to an increase in MAP. Given the evidence of heightened chemoreflexes in HF, we hypothesized that CB activation would result in greater increases in MAP.

Contrary to our hypothesis, activation of the CB led to similar increases in MAP in the HF group compared to the control group. It is known that sympathetic drive to various organs is elevated in HF (Grassi et al., 1995; Sun et al., 1999; Del Rio et al., 2013; Xing et al., 2014; Ramchandra et al., 2018) and this may be one explanation as to why CB activation did not increase MAP to higher levels in HF. We do not have recordings of cardiac SNA in this group of animals so are unable to determine if there are elevated SNA responses to CB activation in HF. It is also important to note that in our study only one CB was being stimulated and it may be that stimulation of both CBs may uncover greater pressor responses in the HF group.

There was no significant difference in the effects of KCN activation on ventilation between the two groups. It is important to note that we are examining the inspiratory effort from the dEMG signal and not tidal volume *per se* so we cannot comment on whether tidal volumes may be different between the two groups.

### The CoBF Responses to CB Activation in Control and HF Animals

We have previously shown that in conscious normal sheep, CB activation increases directly recorded cardiac SNA and CoBF (Pachen et al., 2020). Importantly, infusion of the non-selective beta-blocker propranolol abolished the increase in CoBF (Pachen et al., 2020). We concluded that the β2-adrenergic vasodilatory actions supersede the alpha receptor vasoconstriction during reflex sympathetic activation and are responsible for the coronary vasodilatation. We hypothesized that CB activation may increase cardiac SNA to a greater extent and hence the coronary flow response would be greater in the HF group. When the CoBF responses to CB activation were compared without accounting for metabolic vasodilation, there were no differences between the two groups (Figure 3). When the heart was paced at a steady rate, we could eliminate the effects of metabolic vasodilation and in this scenario, we observed a significantly enhanced CoBF response during CB activation in the HF sheep. The increase in CoBF and CVC was delayed and remained elevated for a longer time period after CB activation (Figure 4). To the best of our knowledge, this is the first time that the effects of CB activation on CoBF have been examined in a conscious model of HF. Our data indicate that activation of the CB leads to a greater increase in CoBF.

### Effect of Propranolol on Carotid Chemoreceptor Stimulation

It is well established that autonomic imbalance with an increase in sympathetic activity and withdrawal of parasympathetic activity is a common finding in HF (Kaye et al., 1995; Bibevski and Dunlap, 1999). In our present study, intravenous propranolol injection decreased HR in sheep with HF more than in the control group (Table 3). This implies that cardiac SNA in the HF animals is maintaining HR to a greater extent compared to control animals.

Intravenous propranolol infusion caused a significant decrease in basal MAP in both groups, suggesting a small but significant role of sympathetic drive to the heart in maintaining blood pressure in these sheep. We hypothesized that the coronary flow response would be mediated by β2-adrenergic vasodilation similar to the normal animals. Contrary to our hypothesis, blockade of beta-receptors did not alter the coronary vasodilation in the HF sheep indicating the vasodilation is mediated by other mechanisms (Figure 6). It is important to note that propranolol is non-selective and can also cross the blood-brain barrier to act centrally (Street et al., 1979). Hence we cannot rule out that the attenuation of coronary vasodilation seen in the normal animals is due to central actions of propranolol. In addition, propranolol can inhibit CB activity under certain conditions such as hypoglycemia (Thompson et al., 2016) so we also cannot rule out an action of propranolol on the CB.

### Effect of Atropine on Carotid Chemoreceptor Stimulation

Previous papers have shown that carotid chemoreflex stimulation by nicotine causes coronary vasodilation in conscious control animals mediated by parasympathetic cholinergic efferent fibers (Hackett et al., 1972; Shen et al., 1994) although this is to contrast to our previous study (Pachen et al., 2020). In our present study, the increased CoBF in both control and HF sheep in response to CB stimulation were unchanged following intravenous atropine infusion. This implies that CB induced coronary vasodilation in HF sheep is not mediated through a cholinergic pathway involving muscarinic acetylcholine receptors. Interestingly, the pressor response to CB activation was attenuated post atropine in the HF group of animals similar to the control animals as we (Chang et al., 2020; Pachen et al., 2020) have previously shown. The site of action of atropine is not clear; however, given its ability to cross the blood-brain barrier (Proakis and Harris, 1979) we presume that atropine may act on the central nervous system within the nucleus tractus solitarius (NTS) (Furuya et al., 2014) and rostral ventrolateral medulla (RVLM) (Padley et al., 2007; Vieira et al., 2007). In addition, acetylcholine (ACh) is released from glomus cells during CB activation to act as an excitatory neurotransmitter (Eyzaguirre et al., 1990; Nurse and Piskuric, 2013) so we cannot rule out a role of atropine on the CB itself.

In the group of animals with HF, neither sympathetic blockade nor parasympathetic blockade altered the increase in CoBF seen during CB activation. As such, the mechanism of the increase in CoBF remains unclear. One possibility is that the parasympathetic nerves have been shown to play a coronary vasodilatory role via other neurotransmitters such as vasoactive intestinal peptide (Feliciano and Henning, 1998). We cannot rule out that the parasympathetic nerves may still play a role but mediated via neurotransmitters other than acetylcholine.

In summary, our findings indicate that in sheep with HF, CB activation causes an increase in CoBF and CVC which is augmented compared to the response in normal animals. Interestingly, the increase in both CoBF and CVC was not mediated by sympathetic pathways, in contrast to normal animals where the increase in CoBF is mediated by an increase in cardiac SNA. Our data indicate that in an ovine model of HF, the CB plays an important role in maintaining blood supply to the heart. Further studies are required to explain the possible mechanisms responsible for CB activation induced increases in CoBF in HF.

## Data Availability Statement

The raw data supporting the conclusions of this article will be made available by the authors, without undue reservation.

## Ethics Statement

The animal study was reviewed and approved by the University of Auckland Animal Ethics Committee.

## Author Contributions

MP, YA, JS, NL, and RR were responsible for the set-up of the animal models in this study including instrumentation and recovery. MP, YA, and JS collected the data. RR supervised the study. MP completed the data analysis and wrote initial manuscript. All authors contributed in revising the manuscript toward the final version.

## Conflict of Interest

The authors declare that the research was conducted in the absence of any commercial or financial relationships that could be construed as a potential conflict of interest.
